# Does the addition of virtual reality training to a standard program of inpatient rehabilitation improve sitting balance ability and function after stroke? Protocol for a single-blind randomized controlled trial

**DOI:** 10.1186/s12883-016-0563-x

**Published:** 2016-03-31

**Authors:** L. Sheehy, A. Taillon-Hobson, H. Sveistrup, M. Bilodeau, D. Fergusson, D. Levac, H. Finestone

**Affiliations:** School of Rehabilitation Sciences, University of Ottawa, Guindon Hall, 451 Smyth Road, Room 3071, Ottawa, ON K1H 8M5 Canada; Bruyère Research Institute, 43 Bruyère St., Ottawa, ON K1N 5C8 Canada; School of Human Kinetics, University of Ottawa, Montpetit Hall, Room 234, 125 University Private, Ottawa, ON K1N 1A2 Canada; Ottawa Hospital Research Institute, 501 Smyth Rd., Box 201B, Office L1298a, Ottawa, ON K1H 8L6 Canada; Department of Medicine, University of Ottawa, Roger Guindon Hall, 451 Smyth Rd., Ottawa, ON K1H 8M5 Canada; Department of Surgery, University of Ottawa, Roger Guindon Hall, 451 Smyth Rd, Ottawa, ON K1H 8M5 Canada; Department of Epidemiology and Community Medicine, University of Ottawa, Roger Guindon Hall, 451 Smyth Rd, Ottawa, ON K1H 8M5 Canada; Department of Physical Therapy, Movement and Rehabilitation Sciences, Bouvé College of Health Sciences, Northeastern University, 407-C Robinson Hall, 336 Huntington Ave., Boston, MA 02115 USA; Bruyère Continuing Care, 43 Bruyère St., Ottawa, ON K1N 5C8 Canada; Faculty of Medicine, University of Ottawa, 505 Smyth Road, Rm. 1200, Ottawa, ON K1H 8M2 Canada

**Keywords:** Stroke, Virtual reality, Rehabilitation, Therapy, Inpatient, Randomized controlled trial

## Abstract

**Background:**

Sitting ability and function are commonly impaired after stroke. Balance training has been shown to be helpful, but abundant repetitions are required for optimal recovery and patients must be motivated to perform rehabilitation exercises repeatedly to maximize treatment intensity. Virtual reality training (VRT), which allows patients to interact with a virtual environment using computer software and hardware, is enjoyable and may encourage greater repetition of therapeutic exercises. However, the potential for VRT to promote sitting balance has not yet been explored. The objective of this study is to determine if supplemental VRT-based sitting balance exercises improve sitting balance ability and function in stroke rehabilitation inpatients.

**Methods/Design:**

This is a single-site, single-blind, parallel-group randomized control trial. Seventy six stroke rehabilitation inpatients who cannot stand independently for greater than one minute but can sit for at least 20 minutes (including at least one minute without support) are being recruited from a tertiary-care dedicated stroke rehabilitation unit. Participants are randomly allocated to experimental or control groups. Both participate in 10–12 sessions of 30–45 minutes of VRT performed in sitting administered by a single physiotherapist, in addition to their traditional therapy. The experimental group plays five games which challenge sitting balance while the control group plays five games which minimize trunk lean. Outcome measures of sitting balance ability (Function in Sitting Test, Ottawa Sitting Scale, quantitative measures of postural sway) and function (Reaching Performance Scale, Wolf Motor Function Test, quantitative measures of the limits of stability) are administered prior to, immediately following, and one month following the intervention by a second physiotherapist blind to the participant’s group allocation.

**Discussion:**

The treatment of sitting balance post-stroke with VRT has not yet been explored. Results from the current study will provide important evidence for the use of low-cost, accessible VRT as an adjunct intervention to increase sitting balance in lower-functioning patients receiving inpatient rehabilitation. The motivating and enjoyable attributes of VRT may increase exercise dosage, leading to improved function and optimal results from rehabilitation.

**Trial Registration:**

https://clinicaltrials.gov/; Identifier: NCT02285933. Registered 06 November 2014.

Funded by the Heart & Stroke Foundation of Canada and a generous donation from Tony & Elizabeth Graham.

## Background

Forty percent of stroke survivors have moderate to severe impairment and 10 % are so disabled that they require long-term care [1]. Balance is often impaired, and 30 % of patients cannot walk independently 6 months post-stroke. Sitting balance ability (control of static and dynamic sitting posture during self-initiated movements eliciting forward/backward, lateral and rotational weight shifting) and sitting balance function (integration of sitting balance control with the performance of functional tasks such as reaching) are useful prognostic indicators of outcome following stroke [2–4], and are impaired in a significant proportion of stroke patients [5].

A majority of studies of rehabilitation interventions following stroke have focused on treatments to regain standing and walking abilities [6–8]. Individuals with greater stroke-related impairment who have not yet acquired these motor skills are excluded. This limits the focus of research outcomes to higher-level mobility tasks. However, studies which have focused on trunk balance training (task-specific reaching, Bobath treatment, using a pointer attached to the body to touch pictures on a screen, biofeedback for seated weight distribution, trunk muscle strengthening and functional exercises) in inpatient rehabilitation populations have shown an increase in maximum reach distance and reach quality, symmetry of seated weight distribution and sitting balance ability [9–12].

Task-specific therapy (such as repeated reaching, or stepping over objects) is effective to improve sitting and standing balance [9, 13–15]. In order to achieve the greatest recovery, patients must be motivated to perform the exercises repeatedly to provide enough treatment intensity (i.e. therapeutic exercise dosage), as the intensity of training has important implications for recovery after stroke [15–17]. In the words of Veerbeek et al. [15], “more practice is better”. Ontario stroke guidelines suggest that patients in inpatient rehabilitation should have at least 3 hours per day of direct task-specific therapy at least 6 days a week [1]. Virtual reality training (VRT) allows patients to interact with a virtual environment while they perform a specific therapeutic exercise. It is enjoyable [18] and may motivate patients to do more repetitions of their exercises [19], contributing to increased intensity.

Past work in our laboratory showed that standing balance exercises performed with VRT produced additional improvements in gait speed and leg function when added to inpatient rehabilitation [20]. Because of legislative change in Ontario, most stroke rehabilitation inpatients are admitted with a Functional Independence Measure (FIM) score in the range of 40–80 [21]. Individuals within the lower end of this range are usually not able to stand independently and therefore would be excluded from most VRT studies where the goal is to promote independent standing function or ambulation. Higher levels of sitting balance predict higher levels of function at discharge from inpatient rehabilitation [22] and sitting training improves not only sitting balance but standing balance (Berg Balance Scale, peak force under hemiparetic foot) and gait function (Dynamic Gait Index) [9, 11]. To date there have been no studies investigating the impact of VRT to improve sitting balance, an important component of stroke rehabilitation for many patients.

The primary objective of this study is to determine if supplemental sitting balance exercises, administered via VRT, improve the control of sitting balance ability in stroke rehabilitation inpatients. Secondary objectives are to determine: 1) whether VRT improves sitting balance function, 2) whether motivation to engage in exercise affects sitting balance outcome, and 3) the psychosocial impact of the use of VRT as an assistive technology for rehabilitation.

We hypothesize that stroke rehabilitation inpatients who perform supplemental VRT that challenges sitting balance will demonstrate improved sitting balance ability compared to inpatients who perform supplemental VRT that does not challenge sitting balance (control group). Secondary hypotheses are that: 1) stroke rehabilitation inpatients who perform supplemental VRT that challenges sitting balance will demonstrate improved sitting balance function compared with the control group, 2) stroke rehabilitation inpatients who are motivated to exercise will improve sitting balance ability and function more than those who are not motivated to exercise, and 3) stroke rehabilitation inpatients will report psychosocial benefits from the use of VRT as an assistive technology for rehabilitation.

## Methods

### Trial design

This is a prospective single-site, stratified, single-blinded, parallel-group study on the addition of supplemental sitting balance exercises, administered via VRT, for the recovery of sitting balance and function in stroke rehabilitation inpatients. Stratification is based on the FIM score, with blocked and balanced randomization.

### Ethics, consent and permissions

This research is being performed in accordance with the Declaration of Helsinki. Approval has been obtained from the Élisabeth Bruyère Research Institute (M16-14-019) and University of Ottawa (A01-15-03) Research Ethics Boards. Potential participants are informed of study details, including procedures, risks and benefits, confidentiality and the voluntary nature of participation, before signing the consent form.

### Participants

Potential participants are being recruited from the inpatient stroke rehabilitation unit of Élisabeth Bruyère Hospital, a tertiary care teaching hospital associated with Bruyère Continuing Care and the University of Ottawa, located in Ottawa, Ontario, Canada. This stroke unit is the largest dedicated inpatient rehabilitation unit for stroke rehabilitation in the Champlain Local Health Integration Network in Eastern Ontario and admits approximately 200 stroke inpatients a year.

Potential participants are eligible if they 1) have had an ischemic or hemorrhagic stroke in the left or right cortical or subcortical region, 2) cannot stand independently for greater than 1 minute or are unable to stand at all, 3) can sit for at least 20 minutes with or without trunk support and can sit for at least 1 minute without trunk support, and 4) can provide informed consent. Potential participants are not eligible if they 1) have an unstable cardiovascular, respiratory, endocrine, orthopedic or neurological condition that precludes exercise of low to moderate intensity, 2) have vestibular deficits or vertigo, or 3) have had seizure activity in the previous 6 months. Aphasia and apraxia of speech are not absolute exclusion criteria, but potential participants must be deemed able to learn VRT in order to participate. Suitable patients are asked by a member of their circle of care (physiatrist, resident, primary care nurse, rehabilitation health care professional) if they are interested in hearing about research studies and if in agreement, are identified to the recruiting member of the research team. Once the study is detailed to the potential participant (by research team members AT-H or LS) and informed consent is obtained, the participant proceeds to the measurement phase followed by the VRT training sessions.

Sample size was estimated from the primary outcome measure [Function In Sitting Test (FIST)] based on the formula for the difference between two independent means using a two-tailed α of 0.05 and a (1-β) of 0.80. We hypothesized that VRT will improve the mean FIST score from the pre-intervention assessment to the post-intervention assessment by a minimum of 6.5 points, which is the minimal clinically important difference (MCID) [23], with a standard deviation (SD) of 9 points. Gorman et al. [23] studied the use of the FIST on 125 individuals with various diagnoses (64 % stroke, 10 % traumatic brain injury, 10 % cancer/tumor resection, 16 % other) attending inpatient rehabilitation. The SD for the FIST in this sample varied between 15.7 on admission and 6.9 at discharge. Because our sample of only stroke patients is more homogeneous we chose a SD of 9.0. This gives appropriate power with a sample size that is reasonable to recruit within two years. While we believe that our experimental intervention of VRT that challenges sitting balance is superior to our control intervention, we cannot rule out that it may be inferior and have conservatively assumed a two-tailed alternative hypothesis. Considering a multivariate repeated-measures design with two groups and three repetitions, the required sample size is 62 participants (31/group). We expect a non-completion rate of 20 % and require each block to have equal numbers; therefore we have set out to recruit 76 participants in total.

Data on demographics (age, weight and height etc.), details about the stroke (location, size etc.) and comorbidities are recorded on each participant, to describe the sample. These characteristics will also be assessed to ascertain if they explain some of the variability in the outcome measures.

### Randomization and blinding

A permuted blocked and balanced randomization method stratified by pre-admission FIM score (low [≤59] or high [≥60]) is being used to allocate eligible participants to the experimental or control groups. The randomization process is carried out with a web-based randomization system based at a remote coordinating centre. The research study team member who completes the pre-intervention outcome measures (AT-H) enters information about a participant’s eligibility and FIM score, and an email stating the participant’s randomization category is sent to the research study team member delivering the VRT (LS), who is the only person to know group allocation until the participant has completed the entire protocol. Using this method, the study team member who performs the outcome measures is blinded to the participant’s group allocation. Healthcare providers on the stroke rehabilitation unit are similarly blinded to their patients’ group allocation. While participants may potentially know whether they are in the experimental or control group, they are cautioned not to remark on the VRT exercises that they receive to the research study team member performing the outcome measures or to their healthcare providers. Results from the outcome measures will not be revealed to the participants or to the research study team member delivering the VRT until after all recruitment, treatment and assessments have been performed for all 76 participants.

### Interventions

Participants in both groups perform VRT for 30–45 min daily for 10–12 sessions over 2–3 weeks. This timing does not include breaks, so a typical session lasts for 45–60 min. The number of sessions completed and minutes of game time per session is recorded. VRT is provided in addition to participants’ traditional rehabilitation program, which consists of 4 hours of physical therapy, 3 h of exercise supervised by a rehabilitation assistant and 3 h of occupational therapy a week, and up to 3 h of speech language pathology a week as required. The specific therapy provided is at the discretion of the rehabilitation professionals, based on national guidelines. Neuropsychology and social work support are provided as needed. The VRT laboratory is located on the inpatient stroke ward; therefore participants do not have to leave the unit to participate.

**Fig. 1:**
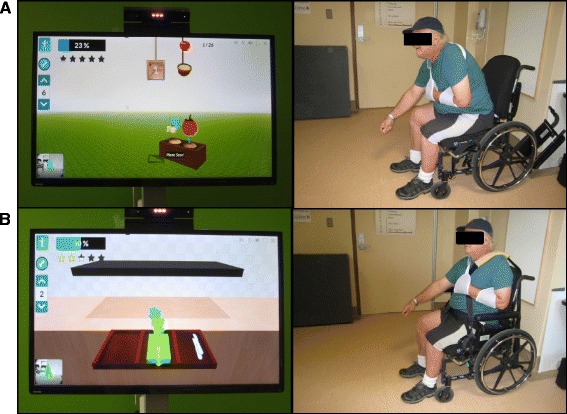
Participant performing virtual reality training using Jintronix Rehabilitation software and a Kinect v2 camera. **a** Intervention game - uses leaning to challenge sitting balance **b** Control game: uses limited arm movements only. Participant is strapped into his chair

VRT is delivered by a single research study team member (LS); a registered physiotherapist with 22 years of experience; 8 years with stroke. She provides constant supervision and monitoring of the VRT, which is delivered using Jintronix Rehabilitation software (Jintronix, Montreal, PQ) and three-dimensional motion capture technology (Kinect v2, Microsoft Canada Co., Mississauga, ON). A camera captures the movements of the participant and allows him or her to control an avatar, which interacts with a game (Fig. 1). The Jintronix system was originally designed for rehabilitation after stroke, is very user-friendly and provides a selection of games/exercises for training sitting balance. Games for the experimental group challenge sitting balance control, reaching and shifting the base of support. Five games are played: Fish Frenzy, Ball Maze, Garden Grab, Bike Barrier and Kitchen Clean-up (Table 1). The difficulty of the games is monitored such that if a participant can perform a game with ease, the level of the game is increased by requiring more speed, distance and/or accuracy in reaching, to continuously challenge the participant’s sitting balance. The Jintronix system gives feedback related to the participants’ performance after each game. A belt is used to secure the upper arm to the trunk to increase trunk lean if necessary. If the game is too challenging or frustrating with respect to sitting balance, the level is decreased, to ensure safety and some success. A CONFORMat™ pressure mat (Tekscan, Inc., South Boston, MA) is used to monitor centre of pressure changes under the buttocks during the intervention. This information assists the research study team member to determine if the game parameters need to be modified to meet the treatment group goals.

**Table 1:** Parameters and movements elicited for each Jintronix game used for experimental and control groups. Flex – flexion, ext – extension, sh – shoulder, horiz – horizontal, add – adduction, abd – abduction, CoP – centre of pressure, rot – rotation, hemi - hemiplegic

Game	Parameters	Movements Elicited	Notes
Experimental Games [no wheelchair arms or seatbelt, sit forwards without back support (if able)]			
Fish Frenzy	Left or right arm; maximum reach (90 %); horizontal plane; reversed figure 8 shape	Trunk flex, elbow flex/ext, sh flex/ext/horiz add/abd	Arm strapped to side to encourage trunk movement
Ball Maze	Equal right & left movements; 100 % of barriers in place on maze	Trunk flex/side flex, encourages greater displacement of CoP	
	Equal right & left movements; 75 % of barriers in place on maze	Trunk flex/side flex, encourages greater precision and control of trunk	
Bike Barrier	30° - 60° trunk side flex, 5 – 10 seconds between movements	Trunk side flex, encourages greater displacement of CoP	
Garden Grab	Left or right arm; Arm drop across body to shin, Arm raise across body to above head	Trunk flex/rot, elbow flex/ext, sh flex/ext/horiz add, encourages displacement of CoP	
Kitchen Clean-up	Left or right arm; maximum (100 %) reach; reaching for cutlery and raising cups	Trunk flex, elbow flex/ext, sh flex/horiz add, encourages displacement of CoP and control of trunk	
Control Games (wheelchair arms in place, seatbelt on, straps crossed across chest)			
Fish Frenzy	Left or right arm; 20 % reach; vertical plane; Δ shape	Elbow flex/ext, sh flex/ext	
Space Race	Left or right arm; 100° range of motion	Sh abd/add	
Catch-Carry-Drop	Bilateral arms; apple falls centre or to hemi side; pipe catches centre or to hemi side; low pipe height; slow or medium speed	Trunk rot (minimal), elbow flex/ext, sh flex/horiz add/abd	Research study team member may assist hemi arm as needed
Kitchen Clean-up	Left or right arm; minimum (20 %) reach; reaching for cutlery	Elbow flex/ext, sh flex/horiz add	
Pop-Clap	Bilateral arms; balloon appears at centre or to hemi side; medium speed	Trunk rot (minimal), elbow flex/ext, sh flex/horiz add/abd	Research study team member may assist hemi arm as needed

The control group spends the same amount of time performing VRT as the experimental group, in order to equalize the additional time spent per week in an engaging activity for both groups. This will allow us to assess the effect of supplemental sitting balance exercises administered with VRT, rather than the effect of VRT per se, on the recovery of sitting balance and function. Control group participants are strapped into their chair to minimize trunk movement [24] and game parameters are chosen to minimize trunk lean and require only limited arm movements. Five games are played: Fish Frenzy, Space Race, Catch-Carry-Drop, Kitchen Clean-up and Pop-Clap (Table 1). Participants in both groups are encouraged to use their more-affected upper extremity; however their less-affected arm is used if the more-affected arm demonstrates poor recovery, fatigue, or pain.

### Outcome measures

**Fig. 2:**
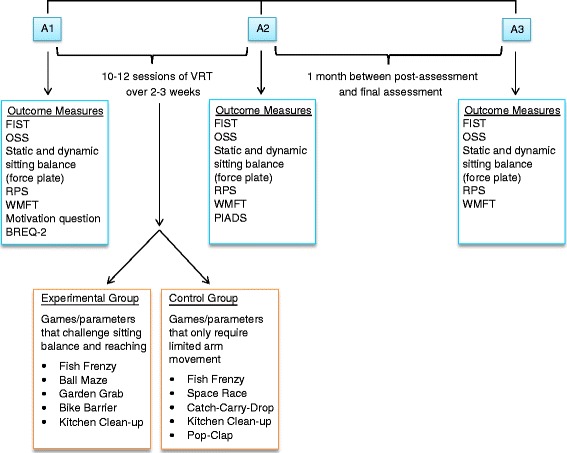
Trial time-line. A1 – pre- assessment; A2 – post- assessment; A3 – 1 month post assessment; VRT – virtual reality training, FIST – Function in Sitting Test, OSS – Ottawa Sitting Scale, RPS – Reaching Performance Scale, WMFT – Wolf Motor Function Test, BREQ-2 – Behavioral Regulation in Exercise Questionnaire, PIADS – Psychosocial Impact of Assistive Devices Scale

Outcome measures are performed by a single research study team member (AT-H, also a physiotherapist, with 28 years of experience), blinded to treatment group. Unless mentioned otherwise, outcomes are assessed before the intervention begins (pre-), at the end of the intervention (post-) and one month later (1 month post-) (Fig. 2).

### Primary objective – sitting balance ability

The primary outcome measure is the Function in Sitting Test (FIST) [25], a 14-item comprehensive test of sitting balance ability and function that includes components of sensory, motor, proactive, reactive and static sitting balance. Each item is scored from zero (complete assistance required) to four (independent). Content, construct and concurrent validity of the FIST have been confirmed [23, 25]. Intra-rater [intraclass correlation coefficient (ICC) 0.99 (95 % CI 0.994 – 0.997)],inter-rater [ICC 0.99 (95 % CI 0.988 – 0.994)] and test-retest reliability [ICC 0.97 (95 % CI 0.847 – 0.995 0] are excellent [26]. Responsiveness of the FIST has also been established, with a MCID during inpatient rehabilitation of 6.5 points, sensitivity of 0.93, specificity of 0.47, an effect size of 0.83 and a standard response mean of 1.04 [23].

Secondary outcome measures include the Ottawa Sitting Scale (OSS) [27] and quantitative measures of static sitting balance [28]. The OSS includes 12 items related to sitting balance ability and function, performed with feet on and off of the floor. Each item is scored from zero (unable, falls) to four (independent). The OSS has excellent intra-rater (ICC 0.99) and inter-rater (ICC 0.96 - 0.98) reliability [27].

To assess static sitting balance participants sit on an AMTI force plate (model 0R6-7-1000, Watertown, MA, US) with their feet off of the floor and positioned such that 75 % of the thigh length is on the force plate. The base of support (BoS) is measured as the area between the greater trochanters, from the front of the force plate to the posterior superior iliac spines. They sit quietly on the force plate for four minutes. Centre of pressure (CoP) displacement is quantified through the creation of an ellipse, normalized to each participant’s BoS. The area of this ellipse represents the participant’s postural sway during quiet sitting.

### Secondary objective – sitting balance function

Three outcome measures are used to address sitting balance function performed during reaching and leaning tasks, the Reaching Performance Scale (RPS) [29], the Wolf Motor Function Test (WMFT) [30] and quantitative measures of dynamic sitting balance. The RPS involves reaching to pick up a cone placed on a table at two distances (1 cm and 30 cm from the front edge of a table placed at arm’s length). Aptitudes such as movement smoothness and trunk displacement are each scored on a scale of 0 to 3, for a total out of 36. Concurrent and discriminant validity have been assessed and preliminary intra-rater and inter-rater reliability are moderate to excellent (ICC > 0.80) [29].

The WMFT assesses the ability to perform 15 upper extremity functional tasks, such as placing the hand onto a table, lifting a pencil and turning a key in a lock. Each item is timed and also scored from zero (does not attempt) to five (normal). Two items also involve strength testing. The WMFT shows good to excellent construct and criterion validity, internal consistency and inter-rater (ICC > 0.88) and test-retest (ICC > 0.90) reliability [31–33]. The minimal detectable change (at the 95 % level, MDC_95_) is 0.7 s for the WMFT Performance Time score and 0.1 points for the average WMFT Functional Ability Scale [34]. The MCID is 19.0 s (dominant side affected) for the Performance Time score and 1.0 points (dominant side) to 1.2 points (non-dominant side) for the Functional Ability Scale [35].

Dynamic sitting balance is quantified to assess the components of postural control relative to the limits of stability (LoS) in sitting [28]. Participants sit on the force plate as before and are asked to lean as far as possible in 8 directions, three times each. An ellipse representative of the LoS is created and normalized to each participant with respect to their BoS. The area of this ellipse represents the extent that a participant can reach without losing stability.

### Secondary objective – motivation to engage in exercise

Motivation to engage in exercise is assessed prior to the intervention (pre-) by asking the question “Over the next 3 weeks, either in the hospital or after you are discharged home, how motivated are you to participate in physical activity for more than 20 minutes for at least 1 day per week?” [36, 37]. The question is repeated with the phrase “… 2 days per week”, etc., up to 7 days per week. Each of the seven versions of the question is scored on a 0 to 6 Likert scale. This measure is reliable (Cronbach’s α 0.92) [38].

The Behavioral Regulation in Exercise Questionnaire (BREQ-2) is also used prior to the intervention (pre-) to assess the quality of motivation for general physical activity [39–41]. The BREQ-2 is a 19-item scale consisting of five subscales, intrinsic motivation (example question, “I engage in physical activity because it’s fun”), identified regulation, introjected regulation (example question, “I feel guilty when I don’t engage in physical activity”), external regulation and amotivation. Each question is scored from zero (not true for me) to four (very true for me). The BREQ-2 has strong factorial validity, acceptable internal consistency and intra-rater reliability (Cronbach’s α > 0.73) [41].

### Secondary objective – psychosocial impact

The 26-item Psychosocial Impact of Assistive Devices Scale (PIADS) is used to measure the effects of assistive devices or technology on “functional independence, well-being and quality of life” [42, 43]. VRT is the technology assessed with this scale, which is administered at the end of the intervention (post-). The PIADS has good internal consistency, construct and predictive validity, high test-retest reliability (Cronbach’s α 0.95) and is responsive to device intervention [42–44].

### Statistical analyses

Baseline characteristics of the participants in the two treatment arms will be assessed using frequency distributions and univariate descriptive statistics including measures of central tendency and dispersion.

To assess the primary outcome measure for the first study objective, a pair-wise analysis evaluating the pre-post and pre-post 1-month intervention mean differences in the FIST with a 95 % confidence interval will be performed. The unadjusted mean difference will be compared first. In addition, a repeated-measures linear regression model will be used to further elucidate the measure of effect while adjusting for possible confounding variables and repeated measures of the FIST. Covariates such as total FIM score, age, and sex will be added to the model using automated stepwise procedures. Variables will be considered for inclusion into the models if there is sufficient statistical evidence and the interaction has clinical rationale. No specific subgroup analyses will be performed.

Analyses of the pre-post and pre-post 1-month intervention mean difference scores (as appropriate) for the OSS, CoP displacement characteristics of the postural sway and LoS ellipses, RPS and WMFT scores will also be reported using mean differences and 95 % confidence intervals. Linear regression modeling of secondary outcomes to assess the impact of various confounding variables (total FIM score, age, sex) will be performed, as for the FIST, above. Scores for the motivation to exercise question, BREQ-2 and PIADS will be compared between groups using t-tests. Scores for the motivation to exercise question will be compared between groups using a Mann–Whitney U test.

## Discussion

This randomized controlled trial has obtained funding from the Heart and Stroke Foundation of Canada (March 2014). Ethics approval was obtained (Nov. 2014) and enrolment began in January, 2015. As of September 2015, 24 participants have been enrolled. Twenty three have completed the post-assessment and 17 have completed the 1 month post-intervention assessment.

The results of this study will provide important evidence for the use of VRT for the recovery of sitting balance in patients post-stroke. Sitting balance is a strong predictor for the recovery of mobility and function [3], however, the effect of VRT on sitting balance as an adjunct activity to inpatient rehabilitation in the non-standing stroke population has not been studied. Most VRT studies have concentrated on higher-functioning patients, to promote standing balance, gait or upper extremity function. For inclusion into this study, a participant need only be able to sit unsupported for one minute, so even some of the lowest-functioning patients, with FIM scores less than 60, can be assessed for a potential benefit from VRT. It is uncommon for stroke patients with this degree of impairment to be involved in research studies.

Repetition of individual exercises is important for the recovery of neural connections/plasticity post-stroke [16]. Each of the 5 Jintronix games used in this study is repeated 30 to 120 times during each VRT session. Over a 10–12 day course of training, each movement is performed up to 1440 times, thereby providing a high intensity of movement repetition. Therefore it is expected that neural recovery could be enhanced using VRT exercise. Positive results from this study could also support the intensification of sitting balance exercises in general for this inpatient rehabilitation population as well as the integration of VRT sitting balance exercises as an integral part of inpatient rehabilitation therapy. The ability to demonstrate that VRT can be provided to highly impaired stroke survivors will be another potential benefit of this study.

We have already shown that VRT for standing balance added to a traditional rehabilitation program provides additional benefits in standing balance and mobility [20]. If the results of this study show that VRT for sitting balance is also effective, a follow-up process would include the integration of VRT sitting balance exercises into this and other inpatient stroke rehabilitation programs. The Jintronix VRT system is relatively inexpensive, simple to learn and easy to customize to individual users. VRT could reasonably be administered by a rehabilitation assistant, as part of patients’ supervised exercise or as an additional therapeutic activity. Supervision by a qualified rehabilitation professional would be ideal in order to avoid undesired movement patterns while performing VRT and to ensure continued pertinence to the patient’s therapeutic needs and goals. There is significant potential for translating the knowledge from this research directly into current treatment practices, such that VRT becomes an integral part of traditional inpatient rehabilitation therapy.

Future research is required on the use of VRT for the treatment of sitting balance by outpatients and in the community. This area of study has not yet been performed but investigation into this area will be important to extend the benefits to the greatest number of stroke survivors while maintaining safety and adherence to treatment goals. VRT has great potential as a treatment modality after stroke; the current study (among others) will help to establish its role.

## Abbreviations

BoS: Base of support
BREQ-2: Behavioral regulation in exercise questionnaire
CI: Confidence interval
FIM: Functional independence measure
FIST: Function in sitting test
ICC: Intraclass correlation coefficient
LoS: Limits of stability
MCID: Minimal clinically important difference
OSS: Ottawa sitting scale
PIADS: Psychosocial impact of assistive devices scale
RPS: Reaching performance scale
SD: Standard deviation
VRT: Virtual reality training
WMFT: Wolf motor function test
